# Multimodal Social Sensing for the Spatio-Temporal Evolution and Assessment of Nature Disasters

**DOI:** 10.3390/s24185889

**Published:** 2024-09-11

**Authors:** Chen Yu, Zhiguo Wang

**Affiliations:** 1 Center for Public Security Technology, University of Electronic Science and Technology of China, Chengdu 610054, China; jlper@uestc.edu.cn; 2 Institute of Public Security, Kashi Institute of Electronics and Information Industry, Kashi 844000, China

**Keywords:** social sensing, multimodal deep learning, disaster management, spatiotemporal analysis

## Abstract

Social sensing, using humans as sensors to collect disaster data, has emerged as a timely, cost-effective, and reliable data source. However, research has focused on the textual data. With advances in information technology, multimodal data such as images and videos are now shared on media platforms, aiding in-depth analysis of social sensing systems. This study proposed an analytical framework to extract disaster-related spatiotemporal information from multimodal social media data. Using a pre-trained multimodal neural network and a location entity recognition model, the framework integrates disaster semantics with spatiotemporal information, enhancing situational awareness. A case study of the April 2024 heavy rain event in Guangdong, China, using Weibo data, demonstrates that multimodal content correlates more strongly with rainfall patterns than textual data alone, offering a dynamic perception of disasters. These findings confirm the utility of multimodal social media data and offer a foundation for future research. The proposed framework offers valuable applications for emergency response, disaster relief, risk assessment, and witness discovery, and presents a viable approach for safety risk monitoring and early warning systems.

## 1. Introduction

Natural disasters are sudden, large-scale public emergencies that cause significant economic damage and human casualties annually, such as floods, earthquakes, and typhoons. In 2021, China experienced 42 heavy rainfall events, affecting 59.01 million people, causing 590 deaths and leading to 245.89 billion yuan in economic losses [1]. It is therefore crucial to quickly and accurately identify potential disaster risks, assess the extent of any damage, and make prompt and effective emergency decisions for minimizing losses and maintaining social stability [2,3]. The collection of disaster information serves as the foundation for the decision-making process [4,5]. Traditionally, disaster information is collected via physical sensors and on-site investigations, but these methods struggle with efficiency, especially during widespread disasters, causing delays in emergency response [6,7]. It is therefore essential that new information sources be developed to address these issues.

In recent years, social media has emerged as a key platform for disaster-related information sharing due to its real-time nature and broad reach [8,9,10,11,12]. Affected individuals express opinions, seek help, or organize mutual aid through these platforms [13,14,15]. Social sensors can build a low-cost, wide-coverage network for efficient information dissemination [16]. Studies on social media usage during disasters, such as the Northern Ireland floods, confirm the volume of mutual aid information shared [17]. The rich personal experiences expressed through social media provide a deeper understanding of disaster impacts, driving the development of disaster semantics and spatiotemporal analysis.

Disaster semantic mining extracts information from both textual and visual social media content using advanced algorithms [13,18,19,20,21]. While text data are highly dense and efficient for dissemination, visual data, through object detection, offers a more intuitive understanding of disaster scenes [22,23,24,25]. Multimodal data help capture the full scope of disaster impacts, making it valuable for emergency management. The number of disaster-related posts also serves as a useful indicator of public attitudes toward disaster events, with post fluctuations reflecting disaster phases and impacts [26,27,28]. However, geolocation data are often limited, presenting challenges for spatial analysis [29]. This highlights the importance of integrating multimodal data with spatiotemporal information in disaster research.

While social media data have advanced disaster informatics, there is room to improve data analysis efficiency, as most existing methods rely solely on textual data [30,31,32,33,34]. Spatiotemporal analysis of multimodal content is crucial for intelligent emergency response. This study introduces a framework that combines text and visual content to extract disaster categories and assess damage severity. The framework uses multimodal neural networks and incorporates location data through named entity recognition (NER), addressing geolocation deficiencies.

A case study on the April 2024 Guangzhou rainstorm demonstrates the framework’s effectiveness in analyzing Weibo data. Key contributions include:

- A disaster analysis framework is proposed for the extraction of disaster-related spatiotemporal information from multimodal social media data. The framework was demonstrated to be an efficient method for analyzing Weibo data pertaining to the Guangdong rainstorm.
- The LLM is used for geolocation pre-processing, whereby supplementary location information is obtained to refine the modelling of the spatiotemporal distribution of disaster-related posts.
- Statistical analysis reveals a strong correlation between the number of disaster-related multimodal posts and precipitation levels. The extracted disaster semantics exhibit a distinctive event-related spatiotemporal distribution pattern. These findings demonstrate that social sensors are able to reflect the varying degrees of impact that disasters have on different regions.

The paper is structured as follows: Section 2 reviews related literature; Section 3 outlines the framework and materials; Section 4 presents statistical analysis results; Section 5 discusses contributions and limitations; and Section 6 concludes with the study’s findings.

## 2. Related Works

With the advancement of information technology and the rise of social media, empirical evidence shows that individuals use these platforms to share and exchange information during crises. Social sensing technology is becoming crucial for gauging humanitarian needs, understanding public sentiment, and monitoring disaster progression. Research in this area is categorized into three main areas: (1) temporal distribution of disaster posts [35], which analyzes the variation in posts and topics throughout disaster phases; (2) spatial distribution of disaster posts, examining the relationship between post locations and disaster impact; and (3) semantic analysis, assessing the public’s reflection on disasters through social media content. While social media timestamps are easily accessible, extracting spatial and semantic information remains challenging.

### 2.1. Spatial Analysis of Disaster Social Media

Geospatial information from social media is vital for spatial analysis and guiding emergency rescue operations [36]. Most studies treat location data as address entities identified via NER models, but only 1–7% of posts have cleaned geolocation tags [37,38,39]. Some studies employ machine learning and natural language processing (NLP) tools for location extraction from text, though they often struggle with unstructured data. Deep learning models show improved performance in mapping words to entities for address extraction [33,40,41]. For example, Yan et al. [29] developed a bidirectional encoder representation from transformers–bidirectional long short-term memory-conditional random fields (BERT-BiLSTM-CRF) model to classify address words. Despite the accuracy of deep learning models on established datasets, real-world social media data’s noise hampers their performance.

Large language models (LLMs) have exhibited considerable ability to generalize across a range of text-based tasks. Their capacity to draw upon a wealth of prior knowledge has also enabled them to effectively identify addresses. Hu et al. [42] used minimal labelled data and geographical knowledge in chat-generative pre-trained transformer (ChatGPT) to extract locations via dialogue. Ambiguity in geographic names complicates location extraction, often requiring additional data for confirmation.

Table 1 compares studies using social media data for spatiotemporal disaster analysis, highlighting the limitations of relying solely on text and check-in data. The unsupervised methods used for disaster category extraction are data-dependent and lack flexibility [26,27,28,43,44,45,46]. Furthermore, studies that employed solely check-in data failed to consider a considerable volume of social media content devoid of check-in tags [26,27,43,44,45,46]. It is crucial to note that the disaster categories extracted through unsupervised methods [26,27,43,44,45,46] are data-dependent and cannot be designed to extract the desired topics. We implemented improvements to address the underutilization of these data.

### 2.2. Semantic Analysis of Disaster Social Media

Social media data are often noisy, complicating the extraction of disaster-related semantics. Modern platforms allow multimodal data (text, image, video), expanding disaster-related insights. Related studies fall into text-based, image-based, and multimodal categories. Text-based studies often focus on public sentiment, using statistical, machine learning, and deep learning methods. Statistical methods focus on keywords [47]. For example, Qian et al. [48] studied the evolution of flood events by analyzing the frequency of occurrence of 17 disaster-related words, such as “warning”, “forecast”, “thunderstorm”, “downpour”, and “flood”. While statistical methods are relatively straightforward to implement, they do not fully leverage the information present in the text. In contrast, numerous studies use machine learning models to extract themes from text. Commonly used machine learning methods include supervised methods such as support vector machine, Naïve Bayes, random forest, logistic regression, and unsupervised methods such as latent Dirichlet allocation (LDA) and K-means [4,26,44,49]. Additionally, deep learning methods are used to encode text sequences, including long short-term memory (LSTM) and bidirectional encoder representations from transformers (BERT), which can incorporate subjectively defined categories for the extraction of specified information [50]. For example, Zhang et al. [51] classified disaster-related elements into distinct categories and employed the NER task to train the BERT model for the extraction of disaster information. Wu et al. [52] used a neural network [53] to investigate the spatial and temporal distribution of climate change-induced affective orientations in microblogging data. Their findings indicate that the majority of Chinese people have a positive attitude towards climate change.

Images provide valuable insights into disaster impact, with visual neural networks learning disaster-related features [22,54,55,56,57,58]. For example, Nia et al. [25] estimated financial damage by analyzing images of damaged buildings, while Li et al. [59] used class activation maps for damage localization. Despite the variability of image data, visual networks like the visual geometry group network (VGG) and residual network (ResNet) have been applied successfully to disaster imagery [60,61].

Combining text and image data in multimodal datasets offers more comprehensive insights than unimodal data alone. Multimodal datasets, though expensive, form the basis for advanced analysis. However, the limited size of these datasets presents challenges for model training. Multimodal models, such as those by Abavisani et al. [62] and Liang et al. [63], integrate features from both text and images and outperform unimodal approaches in tasks like humanitarian classification and damage estimation.

While multimodal data offer richer information, their integration with real-world events is still limited. We propose a new framework for disaster monitoring through social sensing, integrating multimodal content analysis with spatiotemporal analysis.

## 3. Materials and Methods

**Figure 1:**
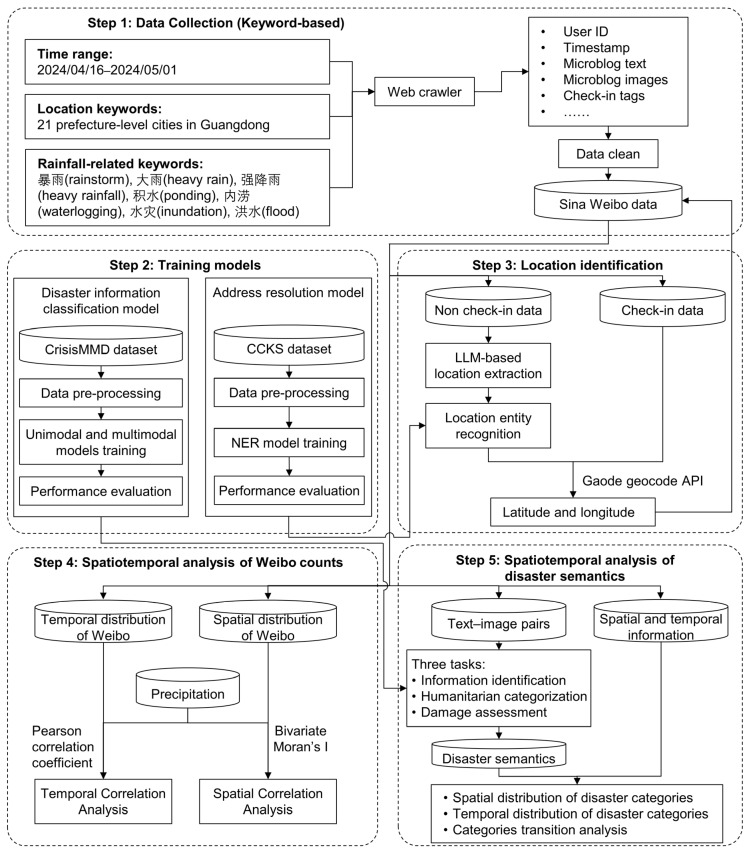
The framework of the proposed social sensing model for spatio-temporal analysis and assessment of disasters for this study.

The workflow of this study is comprised of five steps, as illustrated in Figure 1. The initial step involved the collection of Weibo data based on pre-defined keywords, with the objective of establishing a comprehensive disaster social media database. Secondly, deep neural network models are trained for the extraction of disaster-related information and the recognition of location entities on the publicly available dataset. Thirdly, location information is extracted from Weibo data by LLM and NER models and subsequently encoded into spatial coordinates. Fourthly, the correlation between the number of Weibo posts and precipitation is quantified through quantitative analysis. Finally, the multi-level disaster semantics are extracted using the classification models, and the social perception of the disaster situation is investigated in conjunction with spatiotemporal information.

### 3.1. Study Area

Guangdong province represents the most significant economic centre in the southern region of China, encompassing an area of about 179,700 square kilometres and a population of approximately 127 million. Given its susceptibility to flooding and heavy precipitation, the social sensor in this region is particularly sensitive to rainfall patterns. It can thus reasonably be assumed that the correlation between the multimodal data and rainfall in this region will be statistically significant. In April 2024, sustained heavy rainfall caused extensive damage across numerous regions within Guangdong province. According to data from the Guangdong Meteorological Department, the April average precipitation exceeded the historical records. The heavy rainfall resulted in a series of secondary disasters, including severe urban flooding, river flooding, landslides, house collapses, and road disruptions. These events resulted in a considerable number of casualties and property losses. This particular precipitation event was selected for analysis in this research due to its widespread impact and prolonged impact.

### 3.2. Data Collection

Weibo is a Chinese social media platform with characteristics similar to those of Twitter. It allows users to upload, comment on, and retweet text, images, and video content. The platform has been the subject of numerous studies, which have identified it as a valuable resource. To investigate the impacts of this rainfall event, a corpus of Weibo posts was assembled for analysis. A Python-based web crawler was used to collect data associated with specific keywords via the advanced search functionality of Weibo. In particular, keywords related to rainfall and flooding were selected, including “rainstorm”, “heavy rain”, “heavy rainfall”, “ponding”, “waterlogging”, “inundation”, and “flood”. These keywords were then combined with the names of all prefecture-level cities in Guangdong in order to obtain a series of keyword combinations for retrieval. The data collection period was set from 00:00 on 16 April 2024 to 24:00 on 1 May 2024. The dataset comprised multiple tags and the contents of Weibo posts were downloaded, including post ID, user ID, user name, timestamp, and text content. In the case of posts containing visual content, the images were downloaded in their entirety. Ultimately, a total of 37,010 posts were collected for further processing.

### 3.3. Data Clean

The raw posts contain a considerable number of irregular terms and repetitive content, which impairs the efficiency of content analysis. To enhance the quality of the data, four steps were performed. The text content was initially normalised, whereby uniform resource locators (URLs), special characters, emoticons, and meaningless characters were removed. Secondly, posts with a text length of less than 5 words were considered incomplete and removed. Thirdly, in the case of posts containing duplicate content, one was randomly retained, while the other redundancies were deleted. Fourthly, accounts that solely reposted news and did not contribute original content were removed. Following the aforementioned data-cleaning steps, a total of 20,977 posts were retained providing the necessary materials for the subsequent spatial and temporal distribution study.

### 3.4. Extracting Location Information

The mapping of location information enables the integration of posts into a geospatial space, thereby establishing a foundation for spatiotemporal analyses. The Weibo platform employs two distinct methods for identifying user location: IP-based location tags and check-in tags. The IP location is limited to the provincial level, whereas the check-in tag can be used to identify the POI at the local level. However, it is noteworthy that Weibo posts containing check-in information are relatively rare, comprising approximately 3% of the total dataset. To obtain a greater number of spatial locations, numerous approaches [28,51,64] use NER models to extract location information directly from the text. The capability of NER models to extract location elements depends on the quantity and quality of the training data. Consequently, NER models are unable to make precise predictions when the textual content of Weibo does not align with the expression of the training data. To address the deficiency of location data, a three-stage methodology was used. Firstly, all potential locations within the text were extracted through the use of LLM. Subsequently, further parsing was performed using the NER model. Finally, map services were used to conduct further verification and geocoding of the locations. The LLM was trained on a considerably larger scale than the NER model, thereby enabling more effective noise reduction in the Weibo text. The processed data exhibit enhanced granularity and is better suited to leveraging the capabilities of the NER model.

#### 3.4.1. LLM-Based Geolocation Pre-Processing

LLMs are a class of generative artificial intelligence models that are trained on vast quantities of data with the objective of developing the capacity to comprehend and generate textual content. The Llama 3 model, released by Facebook, is an advanced open-source model that has demonstrated excellent performance on a range of textual tasks. The Llama3 model, which was fine-tuned on Chinese corpora, was employed for the extracting of potential address information from the text. In particular, the model has 8.03 billion parameters with the pre-trained weights from the Ollama library (https://ollama.com/wangshenzhi/llama3-8b-chinese-chat-ollama-q4, accessed on 1 May 2024). The incorporation of extensive prior knowledge in the form of large-scale pre-training data enables the model to effectively mitigate the noise present in non-standard Weibo text.

**Figure 2:**
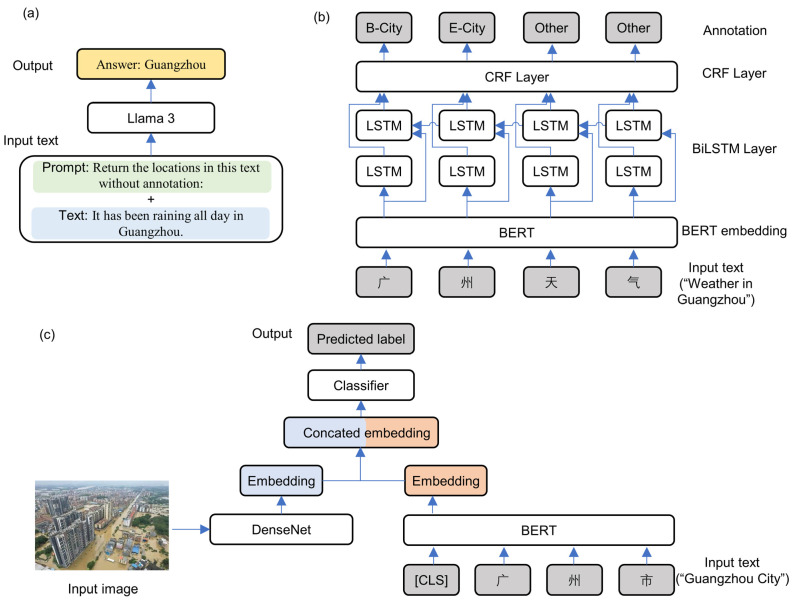
Workflow diagrams for (**a**) Llama3, (**b**) BERT-BiLSTM-CRF, and (**c**) the multimodal models.

Inference is performed by Llama 3 in the form of dialogue, necessitating both texts and prompts as input. The prompts are the text that directs the LLM in the generation of specific content and are directly related to the model output. In order to elicit the desired information, a number of prompts were used, including “This sentence contains location expressions, extract the relevant words” and “Return the locations in this text without annotation”. Figure 2a illustrates the visualization workflow of Llama 3. The absence of a check-in tag in each social media text is indicative of the need to combine these texts with prompts as model inputs for the extraction of location information. It would be optimal for the model to provide a response that includes all of the address elements presented in the input text. Subsequently, all responses were aggregated and de-duplicated. Furthermore, a random selection of Weibo texts from the non-check-in data was used to evaluate the performance of LLM in extracting relevant information. On the test set of 200 samples, the pre-trained LLM achieved a precision rate of 0.99, a recall rate of 0.95, and an F1-score of 0.97. However, the responses invariably included terms that extended beyond mere addresses, as a consequence of the inherent normality of the LLM outputs. Moreover, some datasets include multiple locations with varying geographic levels, which presents challenges in accurately locating them through geocoding services. Therefore, the NER model was used to further normalize these locations.

#### 3.4.2. Location Entity Extraction

The BERT-BiLSTM-CRF model is a frequently used NER model in social media analysis, which is employed for the extraction of geographic entities [28,29]. The model consists of three base models: BERT, BiLSTM, and CRF. BERT is a sequence model based on a self-attention mechanism that encodes words into tokens. The initial parameters for BERT are derived from pre-trained weights that have been trained on Chinese corpora [65], thereby enhancing the efficiency of model training. BiLSTM [66] is a recurrent neural network that is frequently utilized for establishing the contextual relationships between tokens through forward and backward computations. CRF [67] is an undirected graphical model, which can achieve efficient label prediction based on the mutual constraints between tokens. The Chinese address element recognition dataset from the 2021 China Conference on knowledge graph and semantic computing (CCKS) NER challenge [68] was used for the model training. This dataset was divided into 21 geographic levels, such as ‘POI’, ‘City’, ‘District’, ‘Town’, and ‘Road’. The model was trained to extract address nouns by predicting the positions of these labels. The dataset contained 10,826 public address entries and was partitioned into a training set and a test set comprising 8854 and 1972 samples, respectively. The hyper-parameters of the model were determined through a 5-fold cross-validation procedure on the training set. The learning rate was set to 3 × 10^−5^ for BERT and 3 × 10^−3^ for the remaining components. The AdamW optimizer was employed to train the model for 10 epochs. The performance of the model was evaluated using three metrics on the test set: precision, recall, and F1-score, which yielded values of 0.93, 0.92, and 0.92, respectively. The results demonstrated that the model is capable of effectively extracting address information from high-quality data.

The model was employed for the examination of LLM outputs, whereby location information was extracted from the non-check-in data. Figure 2b illustrates the visualization workflow of the BERT-BiLSTM-CRF model. The social media texts were encoded in the form of character sequences by the three models, BERT, BiLSTM and CRF in a sequential manner, thus obtaining annotated sequences. The annotations describe the geographic level (City, Road, Other, and so on) of each character and its position in the word (B-Begin, I-Intermediate, E-End). The aforementioned annotations allow the aggregation of all address terms within the text. Table 2 shows the function of the LLM and NER models through the presentation of several examples. It is evident that the NER model is not an effective means of processing Weibo text. One common issue with the NRR model is the occurrence of inaccurate segmentation, whereby the city “Wuhan” is output alongside the adjective “sunny”. Furthermore, the model occasionally identifies abstract address-related terms as actual addresses, such as “city of laborers”, “six villages”, and “Guangdong road” as observed in these samples. While these phrases are indeed descriptions of addresses, the NER model lacks the capacity to discern the underlying semantics. Consequently, LLM was used to eliminate superfluous terms, thereby enhancing the performance of the NER model. Following the incorporation of LLM, the NER model demonstrated enhanced predictive accuracy.

Table 3 demonstrates the predictive accuracy of the NER model when applied to 200 manually labelled data. The optimal recall is only achieved when all place names are extracted without any irrelevant words. The criterion is more rigorous than that employed in the preceding LLM section, which prioritizes the validation and standardization of the location data. Following the combination of LLM, a notable enhancement in the performance of the NER model was observed, with an increase in recall of 34%. The results demonstrate that the noise in the texts is effectively removed following LLM processing, which facilitates the extraction of locations. In conclusion, the combined model achieves an F1-score of 0.9186, indicating that it has a robust capacity to extract geographical names and is well suited to the objectives of the study.

#### 3.4.3. Geocoding

The locations require additional processing to be transformed into actual geographic locations. The application programming interface (API) of the Gaode map (https://lbs.amap.com/ (accessed on 30 June 2024)) was used to ascertain the latitude and longitude coordinates corresponding to all the place names. Addresses that were not located within Guangdong province were excluded from the subsequent analysis. Additionally, address elements with duplicate names were determined based on the information pertaining to the district, city, and province. Ultimately, a total of 5338 posts with location data were obtained, and the latitude and longitude of these addresses were mapped to QGIS [69] for spatial analysis.

### 3.5. Extracting Disaster Information

The semantics of disasters in social media data are highly heterogeneous, and the mixing of information on different topics reduces their application value [32]. For example, the objective of identifying victims is primarily focused on human-related data, while the objective of disaster reconstruction is centred on damage-related information [70,71]. For this reason, multiple classification tasks were established in a hierarchical structure to facilitate the efficient extraction of disaster-related information. Specifically, three subtasks from the open-source dataset CrisisMMD [72] were designed as strategies for the hierarchical extraction of disaster-related information. Firstly, the collected posts were classified into two categories: “Informative” and “Not informative”. The classification was based on the presence or absence of disaster-related information. Secondly, the collected posts were further classified into a number of fine-grained humanitarian categories based on semantic features. Thirdly, the damage severities were evaluated and classified. The detailed definitions of these categories are in accordance with those set forth in the CrisisMMD dataset. Some categories with approximate meanings have been merged, as shown in Table 4.

#### 3.5.1. Model Training

A two-stream multimodal framework was used for the purpose of conducting a multimodal analysis. In this framework, BERT [65] and DenseNet [73] were used as the text and image encoder, respectively, with the objective of extracting textual and visual features. Figure 2c illustrates the visualization workflow of the multimodal model. The image and text in a social media image–text pair are encoded separately by the encoder of the corresponding modality. The two obtained embeddings are then concatenated to form an overall embedding of the image–text pair. Finally, a linear classifier is employed to make a prediction regarding the disaster category, based on the overall information aforementioned. Since CrisisMMD is in English, the text was translated into Chinese using the Baidu translation API (https://fanyi-api.baidu.com/ (accessed on 30 June 2024)). Text augmentation was achieved through the implementation of the easy data augmentation [74] technique. In order to augment the dataset with regard to the image modality, a series of data augmentation techniques were employed, including random scaling, random cropping, and random flipping. The learning rate was set to 2 × 10^−5^, the optimizer was AdamW, and the number of epochs was 10. To address the issue of class imbalance in the training data and to optimize network weights, focal loss [75] was employed. To evaluate the performance of the model, a series of performance metrics were employed, including precision, recall, F1-score, receiver operating characteristic curve (ROC), and area under the ROC curve (AUC). The ROC curve is a visual representation of the classification ability of a model, demonstrating the relationship between the true positive rate (TPR) and the false positive rate (FPR) at different thresholds.

(1) $$\begin{array}{c} \mathrm{TPR}=\mathrm{TP}/\mathrm{TP}+\mathrm{FN}\\ \mathrm{FPR}=1-\mathrm{TN}/\mathrm{FP}+\mathrm{TN} \end{array}$$

where TP, FP, TN and FN represent the numbers of true-positive, false-positive, true-negative, and false-negative samples, respectively. An ideal classifier should be situated as closely as possible to the upper left corner of the ROC curve, with a false positive rate of 0 and a true positive rate of 1. AUC represents the area under the ROC curve enclosed with the horizontal axes. A value close to 1 indicates the classifier exhibits excellent performance. Furthermore, a unimodal BERT model was trained on the text modality for comparative purposes. A summary of all model and train dataset information is presented in Table 5.

As illustrated in Table 6, the multimodal model exhibits superior performance compared to the unimodal model, achieving F1-scores of 0.9013, 0.8419, and 0.7140 on the three tasks, respectively. 

**Figure 3:**
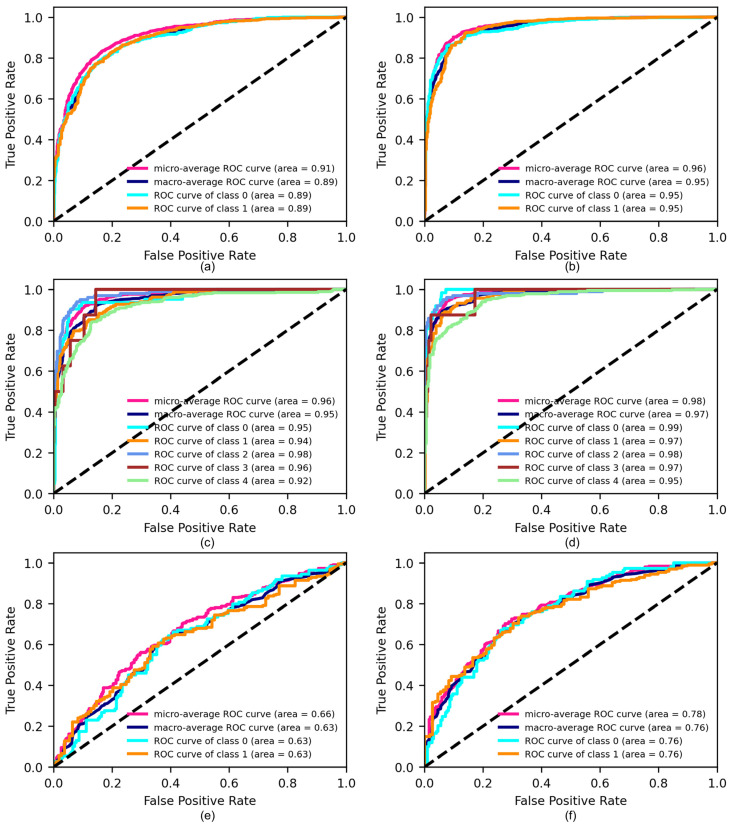
The ROC curves and AUC values of (**a**) unimodal model on task 1, (**b**) multimodal model on task 1, (**c**) unimodal model on task 2, (**d**) multimodal model on task 2, (**e**) unimodal model on task 3, and (**f**) multimodal model on task 3.

These results demonstrate that the multimodal approach leads to more accurate and comprehensive results. The ROC curves and AUC values on the test set are shown in Figure 3. In particular, the multimodal model achieves macro-average AUCs of 0.95 (Figure 3b), 0.97 (Figure 3d), and 0.76 (Figure 3f) on the three tasks, which are superior to the values of 0.89 (Figure 3a), 0.95 (Figure 3c), and 0.63 (Figure 3e) achieved by the unimodal model, respectively. In summary, the multimodal model demonstrates sufficient accuracy in disaster social media analysis. The superiority of the multimodal model across all metrics demonstrates that the incorporation of the image modality offers valuable supplementary information, thus corroborating the hypothesis that multimodal approaches are more effective in this context. The extracted disaster semantics serve as the foundation for spatiotemporal semantic analyses, which are conducted in conjunction with time and location data.

#### 3.5.2. Prediction

**Figure 4:**
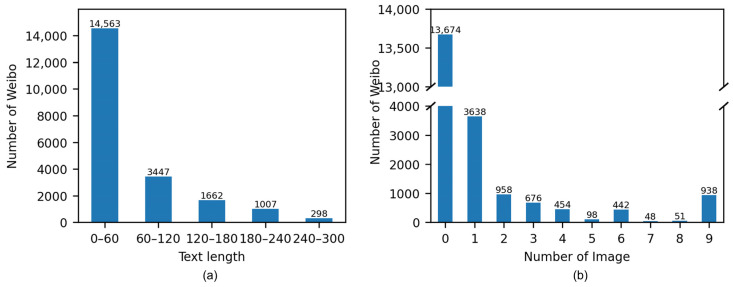
Distributions of (**a**) text length and (**b**) number of images for the collected Weibo data.

The trained multimodal model is used to predict disaster categories from Weibo posts. Prior to this, it is necessary to undertake preprocessing in order to address the discrepancies between the Weibo data and the training dataset. As illustrated in Figure 4a, the length of Weibo texts is considerably longer than that of the training dataset. The maximum length of the training dataset is approximately 60, yet there are numerous Weibo texts that exceed this length. To mitigate this discrepancy, the Weibo texts were segmented into shorter sentences with punctuation marks with a maximum length of 60. In addition, some Weibo posts contain multiple images, while the multimodal model is unable to process. The distribution of the number of images in Weibo posts is shown in Figure 4b, which depicts 7303 (7303 = 3638 + 958 + 676 + 454 + 98 + 442 + 48 + 51 + 938) multimodal posts and 13,674 unimodal posts. The number of Weibo posts with multiple images (3665 = 7303 − 3638) is approximately equivalent to that of posts with one image (3638), and thus cannot be disregarded. In order to utilize all images, each image in the sample was paired with each text segmentation. Subsequently, a max pooling operation was employed to fuse the aforementioned paired features for the purpose of prediction. Similarly, 200 multimodal Weibo posts were randomly selected and manually classified to serve as the test set. The results for precision, recall, F1-score, and AUC are listed in Table 7. The F1 scores for the three tasks are 0.8713, 0.8796, and 0.7808, with AUC values of 0.9339, 0.9219 and 0.7102, respectively. These results suggest that the classification outcomes accurately represent the multimodal content.

Table 8 presents the results of the predictive analysis on a number of samples, with the objective of illustrating the various disaster categories. The first sample mentions the phrase “heavy rain”, but its content is not related to the specified rainfall event. Consequently, it is classified as “Not informative” in the informativeness task. Conversely, the second sample mentions that the rainfall occurs on the highway, therefore it is designated as “Informative”. The image in the third sample depicts a flooded street, thus the predicted label for this sample in the humanitarian categorization task is “Infrastructure, utility, or vehicle damage”, while the label in the damage assessment task is “Severe damage”. The image and accompanying text in the fourth sample pertain to the act of donating and thus are classified as “Rescue, volunteering, or donation effort”. The image in the fifth sample lacks pertinent content; however, the accompanying text describes the casualty status, thus the label in the second task is “Affected individuals”. The sixth sample is related to weather forecasting, and thus its information type is designated as “Other relevant information”. In general, the multimodal models trained with different tasks can effectively extract disaster-related information from social media posts.

### 3.6. Weibo Activity

The demographic characteristics of Guangdong province, including population size and age distribution contribute to the inconsistency in the number of Weibo users across the region. This spatial discrepancy leads to a greater number of Weibo posts in areas with a higher concentration of Weibo users, which in turn affects the effectiveness of Weibo as a disaster indicator. Therefore, the relative number of Weibo (*R_Weibo_*) is employed as a metric for gauging social media activity, which is defined as follows:

(2) $$R_{Weibo}=N_{Weibo}/\sum Population_{i,j}\times Weight_{i,j}$$

where *N_Weibo_* is the total number of Weibo posts in a prefecture-level city, *i* belongs to the age group {0–10, 10–20, 20–30, 30–40, 40–50, 50–60, 60–More}, *j* belongs to the gender group {male, female}, *Population_i,j_* is the population of a specific age *i* and gender *j* [76], and *Weight_i,j_* is the ratio of users corresponding to age *i* with gender *j* [77]. The relative number of Weibo users is stratified by age and gender across a range of geographic regions, allowing for the suppression of spurious spatial characteristics that are dominated by the majority population.

## 4. Results

### 4.1. Spatiotemporal Characteristics of Disaster-Related Weibo

#### 4.1.1. Temporal Distribution of Weibo

**Figure 5:**
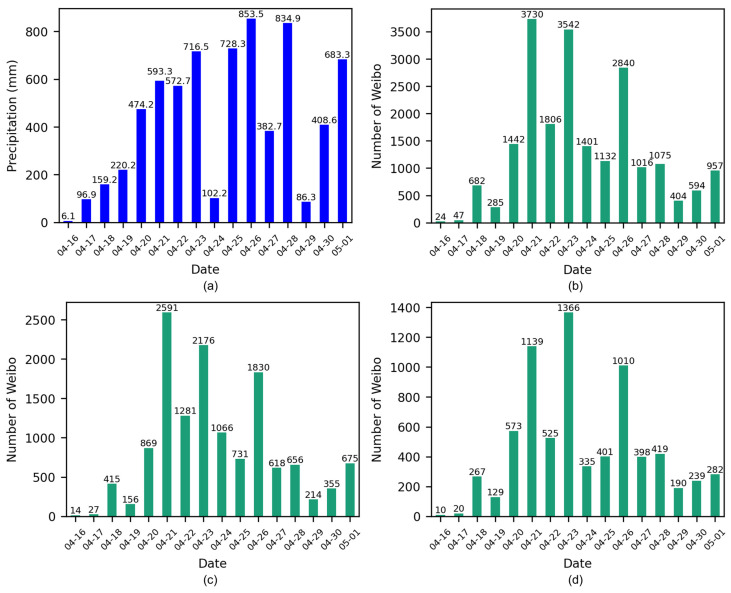
Daily distributions of (**a**) precipitation, (**b**) the total number of Weibo posts, (**c**) the number of unimodal Weibo posts, and (**d**) the number of multimodal Weibo posts.

To examine the perception of heavy rainfall by social sensors, the number of daily Weibo posts from 16 April to 1 May was quantified. To quantify the severity of the disaster, daily rainfall data were collected and are presented in Figure 5a. From 16 April to 19th, the study area experienced minimal rainfall, resulting in a relatively low number of Weibo posts (Figure 5b). The precipitation on 20 April was twice as high as that of the previous day, and the number of Weibo posts also doubled, indicating a notable public response to the rainfall. From 20 April to 28 April, continuous rainfall caused severe damage, resulting in a sustained period of high Weibo activity with daily posts consistently exceeding 1000. Then, a further round of precipitation from 21 April to 1 May was accompanied by a rise in the number of Weibo posts. The peak in the number of Weibo posts occurs earlier than the peak in precipitation, which may indicate a waning public interest in the ongoing rainfall topic. The fluctuations in the number of Weibo posts largely reflected the patterns of precipitation, with the exception of 19 April and 25 April. To quantify the consistency of this relationship, a correlation test was performed using the Pearson correlation coefficient (PCC). As evidenced in Table 9, the correlation between the number of Weibo and precipitation is 0.6367, with a *p*-value less than 0.01, indicating a strong correlation. This result demonstrates the reliability of Weibo data as a research object.

The daily number of unimodal (Figure 5c) and multimodal posts were further counted (Figure 5d) and the correlations with precipitation were presented in Table 9. A correlation coefficient of 0.6125 was observed between the number of unimodal Weibo posts and precipitation, with a *p*-value less than 0.05. In contrast, the result for the multimodal data is 0.6649 with a *p*-value less than 0.01. These findings suggest that the multimodal data exhibit a stronger correlation with precipitation and is more effective in reflecting rainfall patterns compared to unimodal data.

#### 4.1.2. Spatial Distribution of Weibo

A total of 1482 addresses within Guangdong province were obtained from the 1797 Weibo posts containing check-in information. Of these, 519 were identified as non-duplicates. A total of 19,180 non-check-in Weibo posts were examined, resulting in the extraction of 3856 addresses, 294 of which were unique. The non-check-in data provide a substantial number of locations, rendering it a valuable data source for spatial analysis. In the dataset comprising check-in posts within Guangdong province, the number of multimodal posts (932) exceeds the number of unimodal posts (550). This implies that users frequently capture images when checking in, which enables the observation of the disaster scene. In the non-check-in dataset, the number of unimodal posts (2448) exceeds the number of multimodal posts (1408) due to the higher quantity of unimodal data. In total, there are 2340 multimodal locations and 2998 unimodal locations, which demonstrates that multimodal data are also a significant subject for spatial analysis.

**Figure 6:**
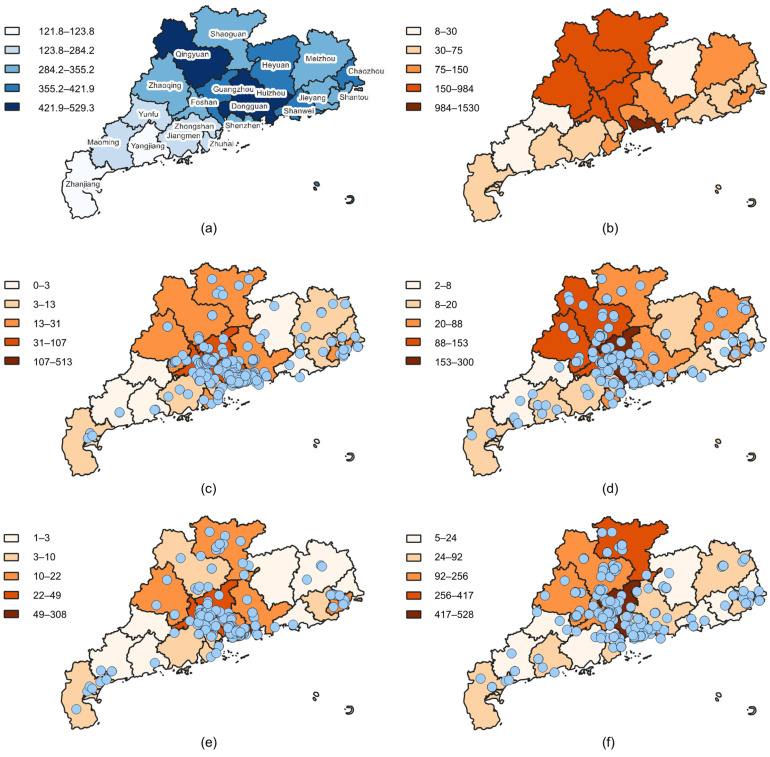
Spatial distributions of (**a**) precipitation, (**b**) the total number of Weibo posts, (**c**) the number of multimodal check-in posts, (**d**) the number of unimodal check-in posts, (**e**) the number of multimodal non-check-in posts, and (**f**) the number of unimodal non-check-in posts. The blue dots represent the actual Weibo address.

The effects of heavy rainfall vary across different regions, leading to disparate responses from social media users. Consequently, data regarding the precipitation and the number of Weibo posts at the prefecture level were gathered. Figure 6a illustrates the total precipitation across Guangdong province from 16 April to 1st May, demonstrating that the majority of rainfall occurred in the central region, with significantly less in the eastern and western areas. Figure 6b presents a count of the number of Weibo posts in each region. It is notable that the distribution of Weibo activity exhibits a pattern that is similar to that observed in the precipitation distribution. Additionally, the distributions of check-in and non-check-in data for both unimodal and multimodal sources are further analyzed. Figure 6c,e present that the check-in data are concentrated in the central and southern regions. The multimodal data provide additional insight into the Meizhou and Chaozhou areas, which is less evident in the unimodal data. The non-check-in data presented in Figure 6d,f provide supplementary information in Jiangmen, Heyuan, and Shanwei. The spatial distribution of rainfall-related Weibo posts exhibits a clustering pattern that is related to precipitation, rather than a random distribution that lacks meaningful coherence.

The region with the highest number of Weibo posts is Shenzhen, yet it is not the region with the highest precipitation. This can be attributed to the number of users on the platform. The age distribution of Weibo users in 2020 indicates that the majority of users are individuals under the age of 30, representing nearly 80% of the total users. Shenzhen has the largest population of young people in Guangdong province, which results in a higher number of Weibo users compared to other regions. To address the issue of concentrated disaster information in densely populated areas, the relative number of Weibo (Equation (2)) was used as an indicator of Weibo activity. The global bivariate Moran’s I can be used to quantify the spatial dependence between two variables. In this study, it is used to measure the correlation between disaster-related Weibo activities and rainfall in Guangdong Province. A Moran value exceeding zero indicates a positive spatial correlation. Conversely, a negative spatial correlation is indicated by a Moran value less than zero. The bivariate Moran’s I in GeoDa [78] was used to quantify the spatial distribution correlation, with the results shown in Table 10. The Moran’s I for the quantity of Weibo data and precipitation is 0.365, with a high level of significance (*p* < 0.01, Z = 3.1154). The results indicate a significant positive spatial correlation between the quantity of Weibo data and precipitation. Furthermore, the non-check-in data exhibit a correlation of 0.336 with precipitation, which is identical to that observed in the check-in data. This suggests that the proposed location extraction strategy yields accurate location data and can provide spatial analyses comparable to those derived from check-in locations. Furthermore, the total, non-check-in, and check-in coefficients of multimodal data are 0.397, 0.340, and 0.346, respectively, which are higher than those of unimodal data (0.339, 0.327, and 0.310). This suggests that multimodal data correlate more strongly with precipitation than unimodal data and are more effective at detecting disasters in space.

### 4.2. Spatiotemporal Characteristics of Disaster Categories

#### 4.2.1. Proportion of Different Disaster Categories

**Figure 7:**
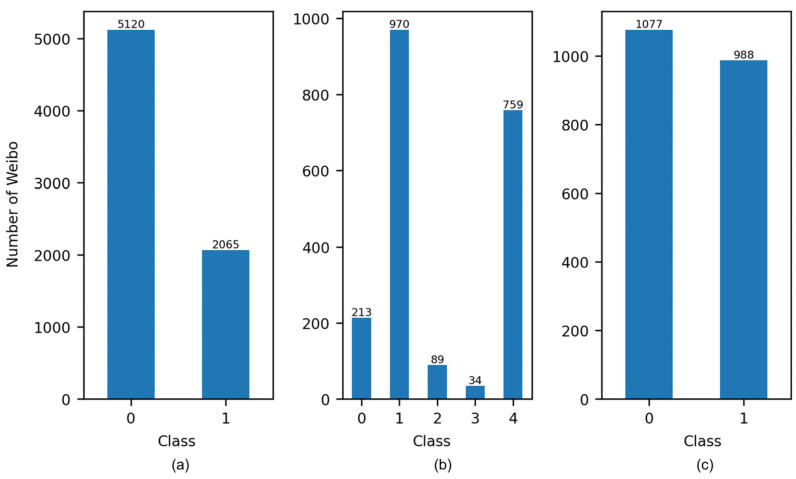
Number of different categories in (**a**) task 1, (**b**) task 2, and (**c**) task 3.

The proportions of disaster-related categories were obtained through the application of the classification models to the dataset. With regard to Task 1 (Figure 7a), the largest proportion is “Not informative”, with informative samples accounting for less than 30%. Figure 7b illustrates the proportion of informative posts within the humanitarian categories. A total of 70% or more of the samples contain humanitarian content, with the largest number of samples falling within the “Other relevant information” category and the smallest number of samples falling within the “Affected individuals” category. This proportion is associated with the characteristics of heavy rainfall. The impacts of persistent precipitation accrue gradually, resulting in the continued accumulation of weather-related data within the “Other relevant information” category. Furthermore, individuals receive warning notifications and proactively avoid flood-prone areas, resulting in a reduction in observations of affected individuals. Figure 7c depicts the damage estimation for informative posts, with approximately half of them classified as severe damage. This suggests that there is a public awareness of the potential for disasters and that social sensors can be used as a means of monitoring the emergence of new risks.

#### 4.2.2. Temporal Statistical Analysis of Disaster Categories

**Figure 8:**
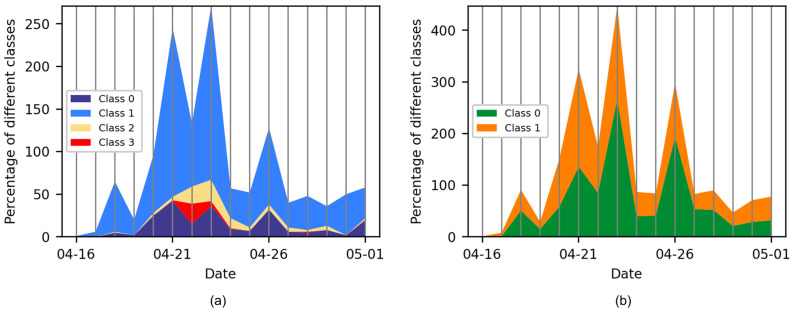
Daily varying counts of disaster categories in (**a**) task 2 and (**b**) task 3.

The daily number of Weibo posts pertaining to various humanitarian and damage categories is presented in Figure 8a,b. For the purpose of qualitative analysis, only those categories deemed relevant are retained. It is evident that the activity of each category is closely correlated with the progression of heavy rainfall. In the period preceding the disaster, up until 19 April, the predominant categories were “Other relevant information” and “Mild, little, or no damage”. As the precipitation levels increased and persisted from 20 April to 23 April, the number of “Infrastructure, utility, or vehicle damage”, “Rescue, volunteering, or donation effort”, “Affected individuals” in Figure 8a and “Severe damage” in Figure 8b exhibited a notable increase. This illustrates that the public is attempting to disseminate disaster-related information and seek assistance through social media platforms. The proportion of the “Affected individuals” category reached its peak on 22 April, coinciding with the first official report of fatalities [79]. Concurrently, the “Rescue, volunteering, or donation effort” category exhibited an increase from 22 April to 23 April, reflecting the implementation of timely emergency relief actions in response to the rainfall that occurred on 21 April. The precipitation ceased for a brief period on 24 April, resulting in a notable reduction in the number of Weibo posts. Subsequently, another period of sustained precipitation commenced on 25 April. The highest precipitation levels were recorded on 26 April, yet the number of damaged posts remained relatively low in comparison to previous observations. This can be attributed to the fact that the public took the initiative to implement preventive measures based on their experience of a previous rainfall event, thereby, reducing their sensitivity to disaster-related damage. The results demonstrate a strong correlation between the temporal shifts in disaster-related categories and the progression of the disaster. In the aftermath of the disaster, social observations regarding damage and injuries began to emerge.

**Figure 9:**
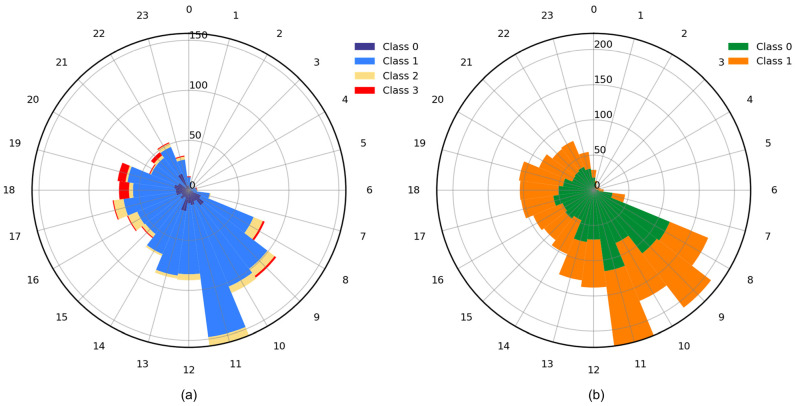
Intraday variation in the counts of disaster categories for (**a**) task 2 and (**b**) task 3.

The intraday distribution characteristics of public perceptions are further examined in Figure 9a,b. To gain insight into the distribution of public opinion throughout the day, all data collected during the study period were counted on an hourly basis. Overall, public activity exhibits a discernible temporal pattern, with activity intensity declining from the morning (6:00–12:00), afternoon (12:00–18:00), to the evening (18:00–24:00), and reaching minimal levels during the early morning hours (0:00–6:00). With regard to the humanitarian categories (Figure 9a), the “Rescue, volunteering, or donation effort” category is concentrated during the daytime, while the “Affected individuals” category is mainly distributed in the evening. This uneven distribution may be attributed to the data source. The “Rescue, volunteering, or donation effort” category posts are from relief organizations, which typically operate during daylight hours and disseminate accordingly. The “Affected individuals” category posts are predominantly sourced from authoritative media, which gather data during the daytime and disseminate the findings in the evening. Regarding the distribution of the “damage” category (Figure 9b), the peak occurs in the morning due to the fact that reports from the previous night are often included in morning news items. Consequently, the number of posts during the morning is approximately twice that of other times of the day. In conclusion, the distribution of daily and hourly messages can assist crisis managers in enhancing the efficacy of their preparation and response strategies. Moreover, the proposed method uses individual image–text pairs as inputs to extract disaster-related information, eliminating the need for a specified number of sample sets. Therefore, the reduced Weibo activity during the early morning hours does not affect the performance of the method.

#### 4.2.3. Spatial Statistical Analysis of Disaster Categories

**Figure 10:**
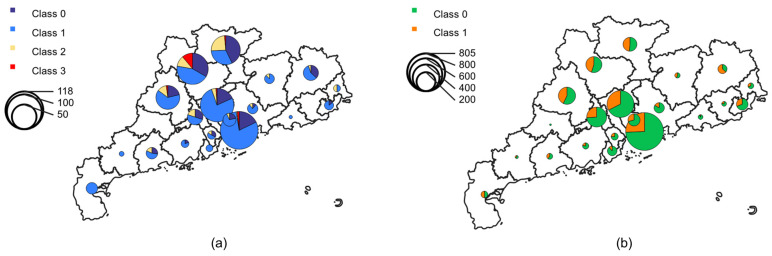
Spatial distributions of disaster categories in (**a**) task 2 and (**b**) task 3.

Figure 10 illustrates the proportion of various categories across different regions. The different colors within the pie chart represent distinct categories, and the size of the circle signifies the quantity of Weibo posts. As illustrated in Figure 10a, the “Other relevant information” category is the most discussed topic across all regions with the exception of two cities. Some cities have posts of the “Infrastructure, utility, or vehicle damage” and “Rescue, volunteering, or donation effort” categories, which are primarily concentrated in the central region. The “Affected individuals” category is more concentrated in the central region, particularly in Qingyuan in the central-northern region. As illustrated in Figure 10b, the majority of cities are predominantly represented by the “Mild, little, or no damage” category, while the “Severe damage” category is concentrated in the central region. Notably, three adjacent cities in the central-northern area, namely Qingyuan, Shaoguan, and Zhaoqing, collectively account for nearly 50% of the “Severe damage” category. These findings support the assertion that central-northern cities are the most severely impacted, which is consistent with the official reports.

#### 4.2.4. Transition of Disaster Categories

**Figure 11:**
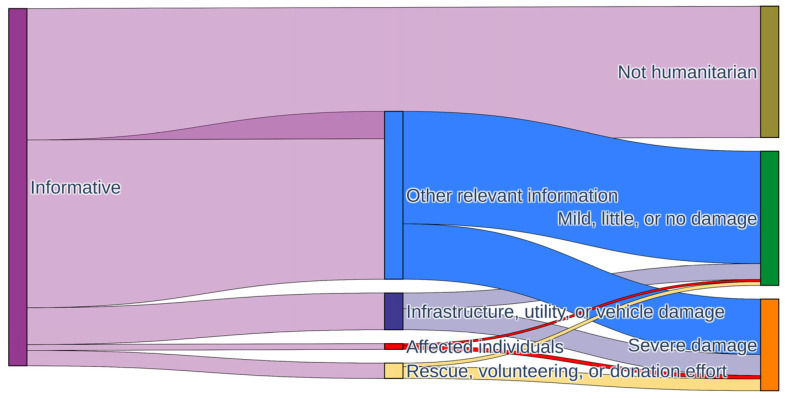
Visualization of semantic transitions across the three tasks.

The study also examines the multiple semantic meanings of samples in order to illustrate the ways in which information varies among the three types of categories. A Sankey diagram is presented in Figure 11 for the visualization of the paths of information flow. The left and right endpoints of each strip indicate the multiple semantics of the corresponding sample subset, and the strip width represents the number of samples in the subset [45]. More than half of the samples in the “Informative” category are transitioned into four specific humanitarian categories, while a relatively small proportion are transitioned into the “Not informative” category. Less than half of “Other relevant information” posts are transitioned to the damage category. In contrast, more than half of the “Infrastructure, utility, or vehicle damage”, “Rescue, volunteering, or donation effort”, and “Affected individuals” posts are transitioned to that category. This indicates that the impacts and damages resulting from rainfall can be described using predefined damage categories. The examination of semantic transitions pertaining to disaster-related topics enables decision-makers to respond effectively to a range of potential hazards.

## 5. Discussion

The examination of actual occurrences within cyberspace can provide invaluable insights for the advancement of disaster research. This study employs a quantitative approach to examine the temporal and spatial correlations between rainfall patterns and Weibo activity in Guangdong province. The findings support the effectiveness of social media can serve as an effective social sensor during this extensive precipitation event. Furthermore, the number of multimodal posts exhibited a slightly higher temporal and spatial correlation with rainfall than the unimodal data, indicating that multimodal data are a valuable research object in disaster informatics. Table 1 presents a comparative analysis of the performance of relevant studies, employing the mean F1-score on the three topic extraction tasks as the evaluation metric. It should be noted that unsupervised models require subjective understanding to specify topic categories and therefore cannot be used to make objective comparisons. It can be seen that the multimodal models outperformed the unimodal model [28,43], which suggests that multimodal applications may offer a promising avenue for future research. The proposed model employed a more advanced visual backbone, which contributed to its superior performance compared to the existing literature [29]. This result highlights the importance of developing advanced models to enhance the efficiency of information extraction.

The analyses of public reactions indicate that in the early phase of the event, public attention was aligned with the onset of the heavy rainfall. The peak in Weibo activity coincides with a peak in precipitation on 21 April, exhibiting minimal temporal lag. However, as the event continues, there is a discernible shift in public attention, which is reflected in a decline in the number of Weibo posts during the subsequent period of rainfall. Furthermore, the shift in various categories demonstrates that messages about rescue efforts and casualties are delayed, whereas posts concerning facility damages and other matters are concurrent with the onset of rainfall. This is due to the fact that the preparation of rescue operations and the gathering of casualty data necessitate a greater investment of time, while other messages are more straightforward to confirm and post. Furthermore, the discrepancy illustrates the real-time character of social media in disseminating disaster-related information. The spatial statistical analysis indicates a notable level of Weibo activity in the affected areas. This suggests that social media can be used to estimate the impact range of disaster events.

Many studies have demonstrated the capability of social sensors in extracting topics, identifying affected areas, and tracking the spatiotemporal evolution of disasters. However, most of these studies [27,28,43,44] focus on mining content from social media to explore the potential of social sensors, with few utilizing statistical methods to characterize the spatiotemporal correlation between rainfall and social media posts. For example, Wang et al. [26] collected rainfall data and Weibo posts during the Zhengzhou rainstorm, analyzing the spatiotemporal evolution of related topics in disaster scenarios. They found that help-seeking messages spiked and then gradually decreased after the disaster, consistent with our findings. However, the lack of quantitative analysis reduces the reliability of using precipitation as a basis for dividing event stages. Wu et al. [45] examined topic transition patterns on social media during heavy rainfall in Hefei. We further characterized the transitions between different thematic categories. Although they presented spatiotemporal distributions for each topic, the absence of statistical indicators makes it difficult to uncover underlying patterns. Yan et al. [29] demonstrated the spatiotemporal distribution of Weibo flood points and rainfall during the Anhui Province rainstorm, confirming the effectiveness of social sensors in revealing affected locations. However, they only analyzed data from two days, limiting insights into the entire event. In contrast, this study quantifies the spatial and temporal correlations between precipitation and social media activity using statistical methods, yielding more compelling results. Although this study makes a notable contribution to the field, it is essential to recognize the limitations of the research. Firstly, the quality of the training data limits the performance of multimodal models. The lack of Chinese multimodal datasets is a significant impediment to the analysis of Chinese social media. Additionally, the majority of commonly used datasets are single-label, yet real-world posts may encompass multiple pieces of information, thereby necessitating the availability of multi-labelled datasets. It is therefore imperative to develop high-quality datasets. Secondly, the current feature fusion methods are not sufficiently interpretable with regard to disaster elements in text and images. This results in the inability to develop targeted emergency response strategies for disaster entities. Accordingly, subsequent research will concentrate on enhancing the interpretability of multimodal models.

## 6. Conclusions

This study examines the effectiveness of multimodal social sensors in enhancing situation awareness in disaster scenarios, with a particular focus on a widespread and sustained rainfall event. A novel framework is proposed for the extraction of multimodal disaster semantics from the Chinese Weibo platform for the purpose of tracking social reflections. Firstly, this study demonstrates that the combination of location extraction methods with large language models can effectively mine address information from text, thereby providing a feasible path for obtaining the spatial distribution from non-check-in data. Secondly, the classification outcomes provide evidence that multimodal models are effective in discerning disaster-related data within authentic social media data, thereby enhancing disaster awareness. Thirdly, the results of the quantitative analysis demonstrate a significant correlation between the quantity of multimodal data and the precipitation level. Furthermore, the correlation between multimodal data and precipitation is stronger than that of unimodal data, indicating that multimodal data are a more reliable source of information regarding disasters. The disaster information extracted by the proposed method from social media platforms can provide support for a number of important emergency response activities, such as emergency rescue, victim discovery, public opinion analysis, post-disaster reconstruction, and other projects during emergencies. In conclusion, these findings substantiate the utility of multimodal social media data in disaster informatics and offer a foundation for future research on multimodal data.

From a practical perspective, future research will focus more on two key areas. Firstly, the development of more high-quality datasets, especially those that are multi-labeled, multi-scenario, and contain more modalities, will enhance the efficiency of models operating with disparate event types. Secondly, an investigation into the interpretability of disaster-related content on social media could provide more direct evidence for the enhancement of emergency response operations.

## Figures and Tables

**Table 1:** Comparison of studies related to the spatio-temporal analysis of disaster-related social media.

Reference	Study Area	Modality	Paradigm	Geo Information	Mean F1-Score
Yan et al. [29]	Province	Text + image	Supervised	Extracted	0.7901
Wang et al. [26]	Province	Text	Unsupervised	Check-in	-
Li et al. [27]	City	Text	Unsupervised	Check-in	-
Peng et al. [28]	City	Text	Supervised	Extracted + Check-in	0.7529
Li et al. [43]	Province	Text	Supervised	Check-in	0.7044
Qu et al. [44]	City	Text	Unsupervised	Check-in	-
Wu et al. [45]	City	Text	Unsupervised	Check-in	-
Karimiziarani et al. [46]	Province	Text	Unsupervised	Check-in	-
This study	Province	Text + image	Supervised	Extracted + Check-in	0.8191

**Table 2:** Examples of location entity extraction from Weibo texts.

Text	NER	NER + LLM
深圳不愧是打工城市,每天到上班这个点都要下个大暴雨 Shenzhen is a city of laborers, and it rains heavily every day when I go to work.	深圳 (Shenzhen), 打工城市 (city of laborers)	深圳 (Shenzhen)
深圳暴雨如注，武汉阳光明媚 Heavy rain in Shenzhen, sunny in Wuhan.	武汉阳光明媚 (sunny in Wuhan), 深圳 (Shenzhen)	武汉 (Wuhan), 深圳 (Shenzhen)
《韶关江湾镇六个村受山体滑坡影响有群众被困，村民：独自在家的母亲仍失联》4月21日，受强降雨影响，韶关市武江区江湾镇遭受洪涝灾害 «Six villages in Jiangwan Town, Shaoguan are affected by landslides and people are trapped» On 21 April, Jiangwan Town, Wujiang District, Shaoguan City, was affected by flooding due to heavy rainfall.	韶关 (Shaoguan), 韶关市 (Shaoguan City), 江湾镇 (Jiangwan Town), 武江区 (Wujiang District), 六个村 (six villages)	韶关 (Shaoguan), 江湾镇 (Jiangwan Town), 武江区 (Wujiang District)
#女生自驾去广东一路暴雨带闪电# # Girls driving to Guangdong all the way in torrential rain and lightning #	广东一路 (Guangdong road)	广东 (Guangdong)

**Table 3:** Performance comparison on location entity recognition.

Model	Precision	Recall	F1-Score
BERT-BiLSTM-CRF	0.9807	0.5250	0.6798
BERT-BiLSTM-CRF + LLM	0.9846	0.8650	0.9186

**Table 4:** Definitions of disaster categories for the three tasks.

Task	Category
Informativeness task (Task 1)	Informative (Class 0) Not informative (Class 1)
Humanitarian categorization task (Task 2)	Infrastructure, utility, or vehicle damage (Class 0)
	Other relevant information (Class 1)
	Rescue, volunteering, or donation effort (Class 2)
	Affected individuals (injured, dead, missing, found) (Class 3)
	Not humanitarian (Class 4)
Damage assessment task (Task 3)	Mild, little, or no damage (Class 0)
	Severe damage (Class 1)

**Table 5:** Detailed information on all models and train datasets.

	Llama 3	BERT-BiLSTM-CRF	Multimodal Model
Data source	Manual labeled	CCKS 2021 NER challenge	CrisisMMD dataset
Data format	Text and label	Text and label	Text–image pair and label
Dataset split	200 (testing)	8854 (training), 1972 (testing)	Task 1: 8814/1101/1103 Task 2: 5503/686/691 Task 3: 2420/302/305
Backbone layers	32 layers	BERT: 12 layers BiLSTM: 2 layers CRF: 1 layer	BERT: 12 layers DenseNet: 121 layers Classifier: 1 layer
Deployment	Pre-trained	Pre-trained + Fine-tuned	Pre-trained + Fine-tuned
Model selection	-	5-fold cross-validation	Hold-out method
Learning rate	-	3 × 10^−5^ for BERT, 3 × 10^−3^ for the rest	2 × 10^−5^
Optimizer	-	AdamW	AdamW
Epochs	-	10	10

**Table 6:** Performance comparison on the three disaster information classification tasks.

Task	Modality	Precision	Recall	F1-Score
1	Multimodal	0.9012	0.9021	0.9013
	Unimodal	0.8315	0.8341	0.8321
2	Multimodal	0.8441	0.8423	0.8419
	Unimodal	0.8181	0.8162	0.8163
3	Multimodal	0.7181	0.7115	0.7140
	Unimodal	0.6106	0.6098	0.6102

**Table 7:** Performance of the multimodal model on real-world Weibo data.

Task	Precision	Recall	F1-Score	AUC
1	0.8821	0.8750	0.8713	0.9339
2	0.8870	0.8850	0.8796	0.9219
3	0.8966	0.7150	0.7808	0.7102

**Table 8:** Some samples and the corresponding predicted labels.

	Texts	Images	Predictions
1	One day in Chaozhou, because of the heavy rain, we played at a friend’s house all day and learnt to play mahjong.	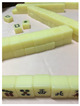	1. Not informative 2. Not humanitarian 3. Mild, little, or no damage
2	Half-day trip to Zhaoqing. I was caught in the biggest rainstorm ever on the highway.	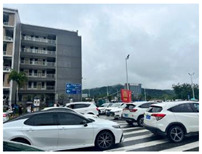	1. Informative 2. Not humanitarian 3. Mild, little, or no damage
3	Aerial photograph of Hanguang town suffering from flooding. Affected by sustained heavy rainfall, Shaoguan and Qingyuan in northern Guangdong province are flooded.	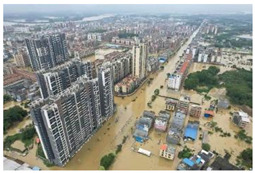	1. Informative 2. Infrastructure, utility, or vehicle damage 3. Severe damage
4	Chinese Red Cross Foundation provides 2000 relief boxes to support flood-stricken areas in Guangdong.	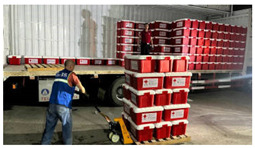	1. Informative 2. Rescue, volunteering, or donation effort 3. Mild, little, or no damage
5	Heavy rains in Guangdong have resulted in 4 deaths and 10 missing. (The meaning of the Chinese characters in the figure is: “News Express @ ChinaNet Live”.)	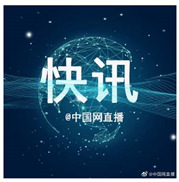	1. Informative 2. Affected individuals 3. Mild, little, or no damage
6	In the past 24 h, the accumulated rainfall in Jieyang has reached torrential levels, and the rain is still ongoing. (The meaning of the Chinese title in the figure is: “Radar image at 42 min on 28 April 2024”.)	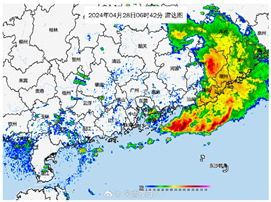	1. Informative 2. Other relevant information 3. Mild, little, or no damage

**Table 9:** Temporal correlations between precipitation and Weibo Counts.

Factor 1	Factor 2	PCC	*p*-Value
Precipitation	Number of Weibo	0.6367	0.0079
	Number of unimodal Weibo	0.6125	0.0116
	Number of multimodal Weibo	0.6649	0.0049

**Table 10:** Spatial correlations between precipitation and Weibo Counts.

Factor 1	Factor 2	Moran’s I	*p*-Value	Z-Score
Precipitation	*Rweibo*	0.365	0.002	3.1154
	*Rweibo* (non-check-in)	0.336	0.003	3.0655
	*Rweibo* (check-in)	0.336	0.003	2.7901
	*Rweibo* (unimodal)	0.339	0.002	2.8922
	*Rweibo* (unimodal, non-check-in)	0.327	0.005	2.7828
	*Rweibo* (unimodal, check-in)	0.310	0.005	2.6523
	*Rweibo* (multimodal)	0.397	0.001	3.4841
	*Rweibo* (multimodal, non-check-in)	0.340	0.001	2.9808
	*Rweibo* (multimodal, check-in)	0.346	0.001	3.0017

## Data Availability

Dataset available on request from the authors.
